# Measuring lip force by oral screens. Part 1: Importance of screen size and individual variability

**DOI:** 10.1002/cre2.63

**Published:** 2017-04-27

**Authors:** Madeleine Wertsén, Manne Stenberg

**Affiliations:** ^1^ Pedodontics and Special Dental Care Sahlgrenska University Hospital Sweden; ^2^ Department of Signals and Systems Chalmers University of Technology Sweden

**Keywords:** drooling, lip force, oral motor dysfunction, oral rehabilitation, oral screen

## Abstract

To reduce drooling and facilitate food transport in rehabilitation of patients with oral motor dysfunction, lip force can be trained using an oral screen. Longitudinal studies evaluating the effect of training require objective methods. The aim of this study was to evaluate a method for measuring lip strength, to investigate normal values and fluctuation of lip force in healthy adults on 1 occasion and over time, to study how the size of the screen affects the force, to evaluate the most appropriate measure of reliability, and to identify force performed in relation to gender. Three different sizes of oral screens were used to measure the lip force for 24 healthy adults on 3 different occasions, during a period of 6 months, using an apparatus based on strain gauge. The maximum lip force as evaluated with this method depends on the area of the screen size. By calculating the projected area of the screen, the lip force could be normalized to an oral screen pressure quantity expressed in kPa, which can be used for comparing measurements from screens with different sizes. Both the mean value and standard deviation were shown to vary between individuals. The study showed no differences regarding gender and only small variation with age. Normal variation over time (months) may be up to 3 times greater than the standard error of measurement at a certain occasion. The lip force increases in relation to the projected area of the screen. No general standard deviation can be assigned to the method and all measurements should be analyzed individually based on oral screen pressure to compensate for different screen sizes.

## 1 INTRODUCTION

Lip force is related to the ability of perioral musculature to produce adequate pressure to tightly close the lips and keep them closed. In the act of swallowing, blowing, sucking, chewing, and pronouncing vowels, the orbicularis oris, buccinators, and superior constrictor muscles function as a unit (Logemann, 1998; Perkins, Blanton, & Biggs, 1977). Lip force is of great importance to remove food from the spoon and to avoid leakage of food and liquid (Chigira, Omoto, Mukai, & Kaneko, 1994). Impaired lip force might cause drooling, retention of food in the vestibulum and affect the swallowing. Apart from being a considerable social handicap, this can be a severe and life‐threatening complication, as aspiration of contaminated saliva in many cases results in pneumonia (Yoneyama et al., 2002). Decreased ability to eliminate food from the oral cavity due to oral muscular dysfunction increases the risk of developing caries. It has been shown that the severity of drooling is positively correlated to sugar clearance time (Gabre, Norrman, & Birkhed, 2005). Drooling and leakage of food from the mouth makes eating with friends and relatives an embarrassing and sometimes even a traumatic experience (Axelsson, Norberg, & Asplund, 1984). Accidental biting of the lip and tongue is reported common in patients with poor oral motor function due to brain damage (Millwood & Fiske, 2001). Furthermore, lip closure is of great importance in articulation when producing bilabial sounds (Barlow & Rath, 1985).

In order to rehabilitate patients with oral motor dysfunction, lip force can be trained using an oral screen which is a curved shield made of acrylic with a handle (Hägg & Anniko, 2008). In the market, there are several different prefabricated oral screens available of different sizes. Training 2 to 3 times a day has been suggested (Thüer & Ingervall, 1990).

Hägg and Sjögreen used a handheld dynamometer, the Lip Force Meter LF 100, and prefabricated oral screens in different material and sizes (Hägg, Olgarsson, & Anniko, 2008; Sjögreen, Lohmander, & Kiliaridis, 2011). Hägg found excellent intra‐investigator reliability testing both patients and controls. Control persons had a significantly stronger lip force than stroke patients using a hard prefabricated oral screen (Hägg et al., 2008). Using a soft oral screen intraindividual variability was tested on healthy adults on two occasions (Sjögreen et al., 2011). The oral screens used in these studies are of different sizes, and thus, it is not possible to compare the measured forces. To our knowledge, whether or not the size of the screen influences on the measured force has not been investigated.

In order to evaluate if the patient improves, fluctuations of lip force in healthy adults must be studied both regarding the variation in one monitoring and how it may change over time. A prefabricated oral screen allows the test person to suck or squeeze during the measuring. Thus, it is uncertain, whether or not it is the force produced by the perioral muscles being measured or if it is a mixture of the force created by sucking and squeezing. This aspect has not been taken into account in any studies. To obtain a reliable measurement of lip force, a method should be selected where the test person squeezes the oral screen without being able to suck.

To express the relative reliability of the measurement, intraclass correlation coefficient (ICC) is a commonly used statistical method. However, a high ICC does not always indicate a small error of measurement in terms of absolute reliability (Atkinson & Nevill, 1998). The value is sensitive to the heterogenicity of the participants. An increasing heterogenicity with a higher standard deviation between subjects and a similar error of measurement will increase the ICC value, thus giving a false impression of accuracy (Atkinson & Nevill, 1998; Hopkins, 2000; Lexell & Downham, 2005). To determine the range of measurement error, the standard error of measurement (*SEM*) and the smallest real difference (*SRD*) should be explored (Beckerman et al., 2001; Lexell & Downham, 2005). A real improvement is shown if the strength increases more than *SRD*.

The aims of this study were to

1. Study how lip force is affected by the size of the screen.
2. Investigate normal values and fluctuation of lip force in healthy adults on one occasion and over time.
3. Identify force in relation to gender.

## 2 MATERIALS AND METHODS

The Ethics Committee of the University of Gothenburg approved the study, (Dnr S43‐96), and it was performed in accordance with the Declaration of Helsinki.

### 2.1 Lip force

The lip force meter LF 100 is an electronic lip force measuring instrument measuring the maximum lip force in Newton over a set period of 10 s (Hägg et al., 2008). A wire is connected to a force transducer based on strain gauge sensing forces from 0 to 250 N with a resolution of 1 N (0.4%). From calibration measurements before and after the test period, the uncorrected bias was less than ±1 N.

### 2.2 Subjects

Twenty‐four healthy adults (12 males and 12 females) were recruited on a voluntary basis (range: 26–73) and informed consent was obtained. The group was mainly composed of dental health personnel at the Public Dental Service. The test persons had ordinary morphology of the face, normal oral motor function, and occlusion. Two males and two females were recruited to each age group. The age groups were 20–29, 30–39, 40–49, 50–59, 60–69 and 70+.

### 2.3 Oral screens

**Figure 1:**
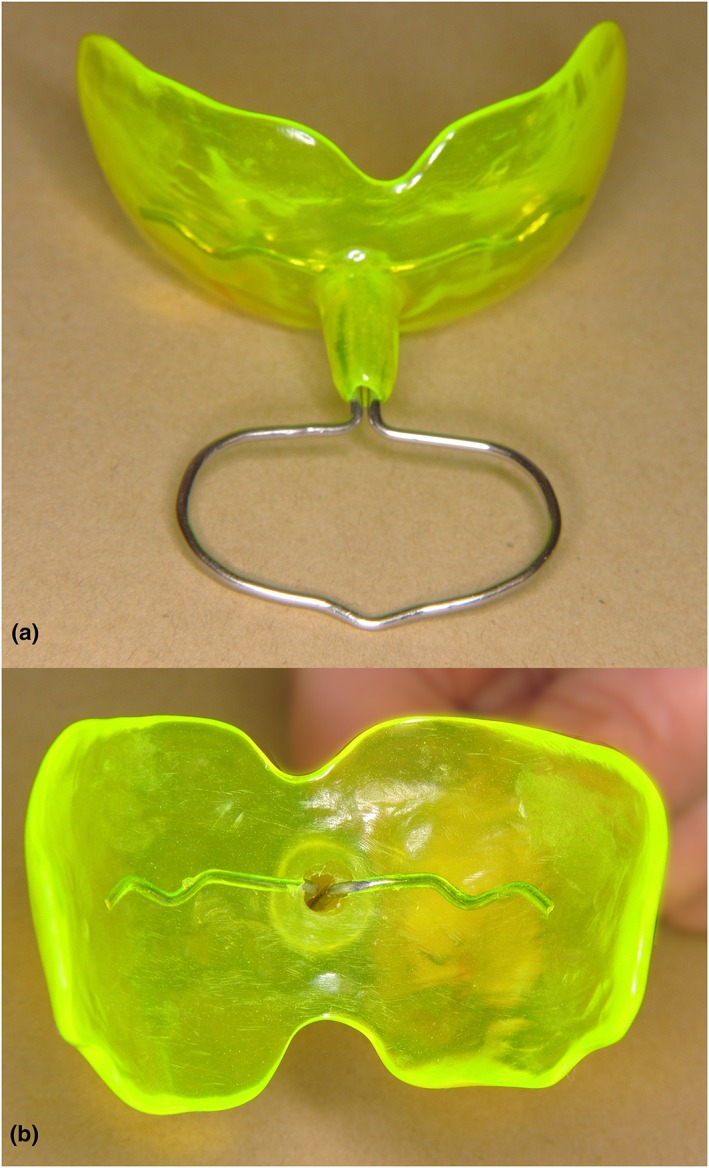
(a) Oral screen with front handle and tube; (b) The hole seen from inside

Three different sizes of oral screens—small, medium, and large—were made from plaster casts measuring 45 mm, 49 mm, and 56 mm between the buccal surfaces of teeth 15 and 25. The oral screens were made of acrylic and covered the oral vestibule in the front and back to the distal surfaces of the second premolars each side. They were designed with a small hollowed tube around the handle (Figure 1a,b). The tube made it possible to let air pass and prevent suction.

### 2.4 Projected area of the oral screen

The screen was placed on a piece of paper. By looking from a perpendicular direction, the parallel projected contour was identified and drawn on the paper. A reference area of known size was applied to the paper. The paper was scanned and analyzed in an image manipulation program (GIMP). The projected area of the small screen was 13.4 cm^2^; the medium, 15.5 cm^2^; and the large, 22.6 cm^2^. The maximum error was estimated to 5% of measured area.

### 2.5 Measurement procedure

The examiner demonstrated the measuring procedure and gave the verbal instruction: “Hold the oral screen in your mouth as firm as you can, while I pull it out.” The screen was placed inside the lips. The wire was stretched perpendicular to an imaginary line between the nose and the chin of the test person, and the measuring was started. The examiner pulled the wire gradually increasing the power until the oral screen was pulled loose. The procedure was repeated 3 times in succession for each screen.

### 2.6 Data collection

The lip force was measured at 3 times during a period of 6 months with 3 months between the measurements. No exercise was to be performed by the participants. The measurement procedure was carried out 3 times for each size of oral screens at each occasion. Changing from one size of the oral screen to another, the test person rested for 2 min. For each of the 24 individuals, 27 measurements were carried out in total. There were 216 measurements for each screen and in total, 648 measurements in the dataset. The same investigator made all measurements.

### 2.7 Statistical analysis

The dataset was analyzed with SPSS and further processed in MS Excel. Calculations of the confidence limits for the standard deviations were based on the χ^2^ distribution. Upper and lower bounds of the standard deviations at 95% confidence level were calculated in MS Excel for different *n* values. Homogeneity of variances was tested in SPSS with Levene's test and one‐way analysis of variance (ANOVA) was used to compare means. The data was analyzed in SPSS for normality by the Shapiro–Wilk test. The difference between men and women was tested with Student's *t* test. One‐way ANOVA was used to test variation over time for different individuals.

Measurements were divided among the three time groups, and an estimated standard deviation within the same occasion (*SEM*) was calculated from a one‐way ANOVA analysis as the square root of the mean square within groups (*MSWG*).

(1) $$\mathrm{SEM}=\sqrt{\text{MSWG}}$$

A normalized quantity *SEM*% can be calculated from the relation:

(2) $$\mathrm{SEM}\%=\left(\frac{\mathrm{SEM}}{\text{mean}}\right)\cdot 100,$$

where *mean* is the mean of all measurements. In order to analyze the magnitude of changes with time, a relative mean value change *d*_*i*1_ was calculated according the following equation:

(3) $$d_{i1}=\frac{{\bar{x}}_{l}-{\bar{x}}_{1}}{\sqrt{\text{MSWG}}}=\frac{{\bar{x}}_{l}-{\bar{x}}_{1}}{\mathrm{SEM}},$$

where

$\bar{x_{i}}=\;$Mean value at time *i*,

$\bar{x_{1}}=\;$Mean value at time 1.

A 95% confidence level of significant difference between two measurements is often calculated according the following relation (Beckerman et al., 2001):

(4) $$\mathrm{SRD}=1.96∙\mathrm{SEM}∙\sqrt{2}$$

In our case we calculate the mean $\bar{x_{i}}$ at every occasion from *m* = 6 measurements. With *k* = 3 occasions, we get in total *n = mk* = 18 measurements for each individual. A 95% confidence level of significant difference between two means could then be calculated as

(5) $${\mathrm{SRD}}_{\text{mean}}=1.96∙\frac{\mathrm{SEM}}{\sqrt{m}}∙\sqrt{2}$$

However, from ANOVA the calculation of the *SEM* value is based on a limited number of measurements with *df* = *n − k* degree of freedom. We must then introduce the *t* statistics for a more accurate calculation of *SRD*_*mean*_ giving

(6) $${\mathrm{SRD}}_{\text{mean}}=t_{.975,\mathrm{df}}∙\frac{\mathrm{SEM}}{\sqrt{m}}∙\sqrt{2}$$

where *t*_.975 , *df*_ is the value of the *t* statistic with cumulative probability .975 and *df*, degrees of freedom. In our case, we get *t*_.975 ,  15_ = 2.13 and

(7) $${\mathrm{SRD}}_{\text{mean}}=2.13∙\frac{\mathrm{SEM}}{\sqrt{6}}∙\sqrt{2}=1.22∙\mathrm{SEM}$$

## 3 RESULTS

### 3.1 Screen size

**Figure 2:**
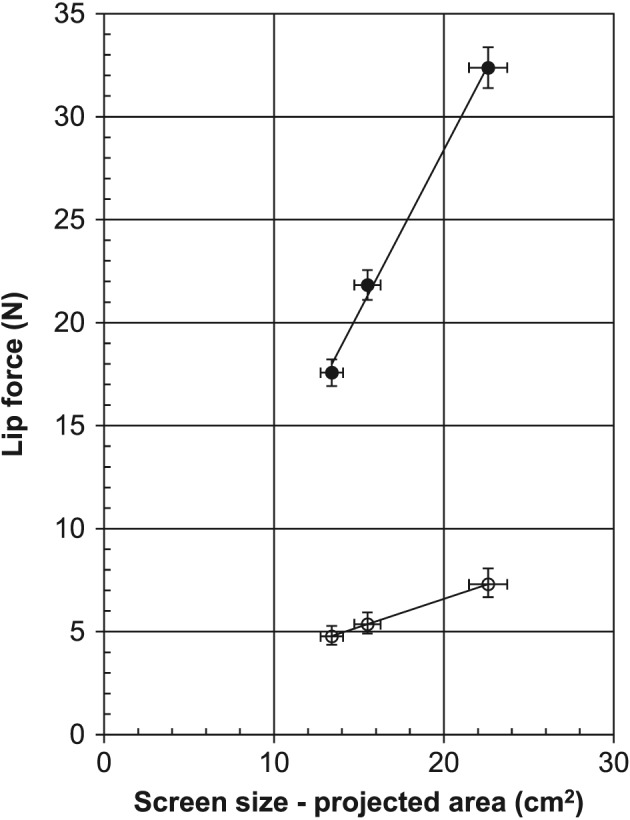
The dependence of screen size for the measurement of lip force. Filled circles (●): Mean value of lip force for all measurements. Open circles (○): Standard deviation for individual measurements. Vertical error bars are 95% confidence limits for mean value and standard deviation. Horizontal bars are showing estimated 5% maximum error in area measurement

An overall picture of the whole dataset is given in Figure 2, where the data is divided between the three different screen sizes. The mean value and standard deviation for single measurements differs between the screen sizes. Error bars are showing the 95% confidence limits for the measured parameters. Calculation of the 95% confidence limits for the standard deviations are based on the χ^2^ distribution (*df* = 215, lower limit 0.91·SD, upper limit 1.10·SD). The mean value of lip force varies significantly with screen size. From a Levene's test, it was concluded that the variances were significant different, *F*(2, 645) = 16.1, *p* < .001. By dividing lip force with the projected area of the screen, a normalized value can be obtained which is independent of the screen size. The new parameter will have the dimension of pressure, that is, force per unit area. The term “oral screen pressure” (OSP) is used for this parameter expressed in kPa (kilopascal), where 1 N/cm^2^ = 10 kPa.

After analyzing the OSP data with Levene's test, it was found that the variances were not significantly different, *F*(2, 645) = 1.06, *p* = .346. The mean value for the smallest screen was significantly smaller than the mean values from the medium screen and large screen (Table 1). However, mean value did not differ significantly between medium and large screens. The data was analyzed in SPSS for normality by the Shapiro–Wilk tests. The measurements might be normally distributed since the *p* values in the Shapiro–Wilk test are greater than .05. However, for the small screen, a deviation from a normal distribution is seen. From these results, it was concluded that measurements from the medium and large screen could be combined in order to analyze individual variability. However, measurements from the small screen could be biased with a small systematic error. Thus, the small screen values were excluded from further variability analyses. The total number of measurements is *N* = 18 for each individual in the following analyses.

**Table 1:** Oral screen pressure data for different screens

Screen size	Projected area (cm^2^)	Mean value (kPa)	Standard deviation (kPa)	95% confidence interval for mean (kPa)	Normality (*p* value, Shapiro–Wilk)
Small	13.4	13.11	3.56	12.63–13.59	<.001
Medium	15.5	14.08	3.46	13.62–14.54	.059
Large	22.6	14.33	3.23	13.89–14.76	.469

### 3.2 Differences between individuals

**Figure 3:**
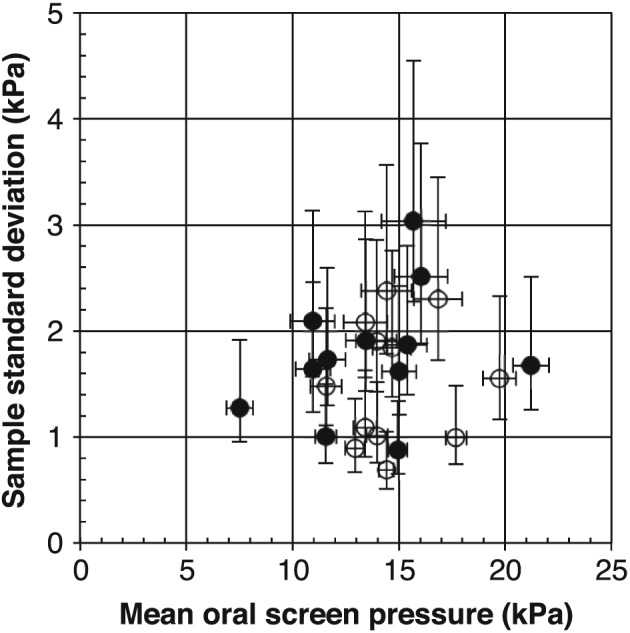
Oral screen pressure data for 24 individuals. Sample standard deviation versus mean value of oral screen pressure (*N* = 18 for each individual). Filled circles (●): Women; open circles (○): Men. Error bars show 95% confidence limits

In Figure 3, OSP standard deviations for single measurements are shown versus mean OSP for each subject. Calculation of the confidence limits for the standard deviations was based on the χ^2^ distribution (*df* = 17, lower limit 0.75·SD, upper limit 1.50·SD). Here, it can be seen that there are major individual variations. Both the mean value and the standard deviation for single measurements are different for the various subjects. Individual mean values are distributed around 14.2 ± 2.9 kPa (mean ± SD).

### 3.3 Variation associated with gender

The OSP values for women were 13.7 ± 3.5 kPa and 14.7 ± 2.3 kPa for men (mean ± SD). An independent samples *t* test showed no significant difference in mean value between men and women, *t*(22) = −0.88, *p* = .39.

### 3.4 Variation over time

A one‐way ANOVA analysis was carried out for each subject in order to investigate possible significant changes in the mean value with time. From Levene's test, it was found that for all individuals except two, the variances were not significantly different at the three different occasions. The ANOVA data could be used to make a more thorough investigation of the spread of mean values and estimated *SEM* values for different subjects (Equation (1)).

**Figure 4:**
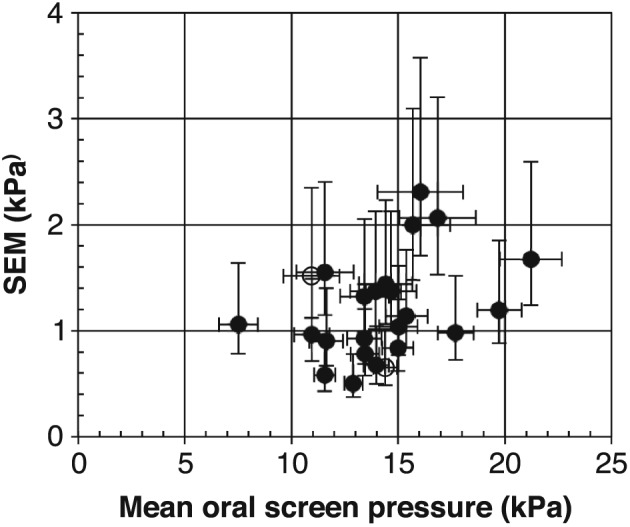
Oral screen pressure data for 24 individuals. Same data as in Figure 3 but now showing estimated standard deviation within the same occasion (*SEM*) based on ANOVA analysis. Filled circles (●): Variances are not significantly different at different occasions. Open circles (○): Variances may be different at different occasions. Error bars show 95% confidence limits

Figure 4 shows a plot of the estimated *SEM* value versus the mean value of OSP for each subject. Calculation of the confidence limits for the *SEM* value was based on the χ^2^ distribution (*df* = 15, lower limit 0.73·SD, upper limit 1.58·SD). The magnitude of the *SEM* value is lower than the previous standard deviation in Figure 3 since the spreading, due to measurements at different occasions, is now eliminated. However, it is seen that there is still a wide spreading in *SEM* values among the subjects indicating that this parameter is really an individual parameter. Individual parameter data are summarized in Table 2. Here, it can be seen that both mean values and *SEM* values may be normally distributed because Shapiro–Wilk test gave *p* values significantly greater than .05. The data in Figure 4 and Table 2 may be converted into values of *SEM*% (Equation (2)). The *SEM*% values were found to be in the range 4%–14% with a mean of 8.6%.

**Table 2:** Oral screen pressure data for different individuals

Parameter	Mean value (kPa)	Standard deviation (kPa)	95% confidence interval for mean (kPa)	Normality (*p* value, Shapiro–Wilk)
Individual mean value	14.2	3.9	13.0–15.5	0.62
*SEM*	1.2	0.48	1.0–1.4	0.30

**Figure 5:**
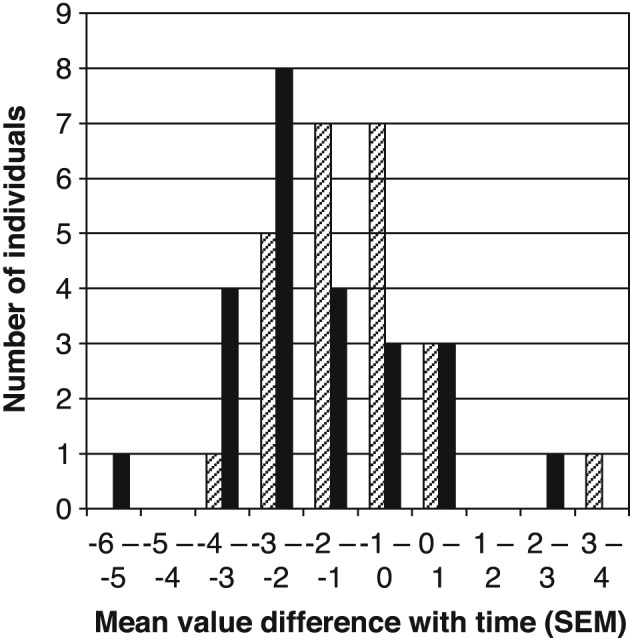
Histogram of the oral screen pressure mean value differences between measurements carried out at different times. Mean value differences are in units of *SEM* (see text). Partially filled bars are changes after 3 months; solid bars are changes after 6 months

Figure 5 shows a histogram of the oral screen pressure mean value differences between measurements carried out at different times (Equation (3)). It can be seen that the mean value often decreased over the time period of 3 to 6 months. The majority of changes were in the interval −4 to +1 *SEM*. Significant variations in mean values were found for changes greater than ±1.22 *SEM* (Equation (7)). Of the 24 subjects, 14 showed significant changes after 3 months, and 17 showed significant changes after 6 months (α level .05).

## 4 DISCUSSION

### 4.1 Size of screen

To produce a sufficient and tight pressure of the lips, several muscles work simultaneously. The muscles form a membrane that exerts pressure on the surface of the oral screen. By converting lip force into OSP, it will be possible to compare studies, where oral screens with different sizes have been used, and thus, it is important to document and present data of oral screen size. The fitted line in Figure 2 crosses the *x* axis at approximately 2 cm^2^. The reason may be that the lips do not touch the area in the middle of the screen, where the handle fits in. Accordingly, no tissue will hit this spot. Consequently, the smaller the screen, the greater the relative importance of this area. Unfortunately, the result from this study cannot be compared with the results from other studies as, to our knowledge, there are very few articles and no literature to refer to in this area. This indicates that there is great need for further research in this field.

### 4.2 Gender

As in other studies, no significant difference regarding sex has been found (Sjögreen et al., 2011). A possible variation associated with gender (around 1.0 kPa) is small compared to the individual variability (standard deviation 2.9 kPa).

### 4.3 Test–retest reliability

It will be difficult to assess an improvement if the values for an individual vary greatly within the same measurement occasion. This study has shown that the intraindividual test–retest reliability varies between individuals. To analyze whether the lip force for a person has improved, a standard of the individual variance has to be calculated. Therefore, it is essential that every study be based on a large number of measurements. By using the mean of three measurements at each of three occasions, we get *SRD*_*mean*_ ≈ 2.0 *SEM* (Equation (7) with *t*_.975 ,  6_ = 2.45). This value may be compared with normal random fluctuations over long time periods which were found to be within ±4 *SEM*, indicating that little is gained by further increasing the number of measurements at each occasion.

The reliability of lip force measurements could be compared with literature data of muscle strength. Measurements on leg have given *SEM*% values in the range 2.1% to 8.2% for different muscle groups (Lu et al., 2011). As these measurements were based on the average of three measurements at every occasion, our data of *SEM*% should be divided by a factor $\sqrt{3}\approx 1.73$ giving a range 2.3 to 8.1 for the different individuals.

### 4.4 Further studies

Oral screens without possibility to mix suction and squeezing have been used in this study. There is a great need for studies to clarify the difference between measuring with and without suction.

## 5 CONCLUSION

1. The maximum lip force depends on the area of the screen size. By evaluating the projected area of the screen, lip force could be normalized to an OSP quantity that can be used for comparing measurements from screens with different sizes.
2. Both the mean value and standard deviation for single measurements were shown to vary between individuals. Therefore, no general standard deviation measure can be assigned to the method and all measurements should be analyzed individually.
3. For a particular individual, longitudinal data can be analyzed by variance analysis (ANOVA).
4. Normal variation over time (months) may be up to 4 times greater than the *SEM* at a certain occasion.
5. No significant relation to gender was found.

## References

[cre263-bib-0001] Atkinson, G. , & Nevill, A. M. (1998). Statistical methods for assessing measurement error (reliability) in variables relevant to sports medicine. Sports Medicine, 26(4), 217–238. 9820922 10.2165/00007256-199826040-00002

[cre263-bib-0002] Axelsson, K. , Norberg, A. , & Asplund, K. (1984). Eating after a stroke—towards an integrated view. International Journal of Nursing Studies, 21(2), 93–99. 6589215 10.1016/0020-7489(84)90050-6

[cre263-bib-0003] Barlow, S. M. , & Rath, E. M. (1985). Maximum voluntary closing forces in the upper and lower lips of humans. Journal of Speech and Hearing Research, 28(3), 373–376. 4046578 10.1044/jshr.2803.373

[cre263-bib-0004] Beckerman, H. , Roebroeck, M. E. , Lankhorst, G. J. , Becher, J. G. , Bezemer, P. D. , & Verbeek, A. L. M. (2001). Smallest real difference, a link between reproducibility and responsiveness. Quality of Life Research, 10, 571–578. 11822790 10.1023/a:1013138911638

[cre263-bib-0005] Chigira, A. , Omoto, K. , Mukai, Y. , & Kaneko, Y. (1994). SummerLip closing pressure in disabled children: A comparison with normal children. Dysphagia, 9(3), 193–198. 7521812 10.1007/BF00341264

[cre263-bib-0006] Gabre, P. , Norrman, C. , & Birkhed, D. (2005). Oral sugar clearance in individuals with oral motor dysfunctions. Caries Research, 39(5), 357–362. 16110206 10.1159/000086841

[cre263-bib-0007] Hägg, M. , & Anniko, M. (2008). Lip muscle training in stroke patients with dysphagia. Acta Otoaryngol., 128(9), 1027–1033. 10.1080/00016480701813814 19086198

[cre263-bib-0008] Hägg, M. , Olgarsson, M. , & Anniko, M. (2008). Reliable lip force measurement in healthy controls and in patients with stroke. A methodological study. Dysphagia, 23(3), 291–296. 18253790 10.1007/s00455-007-9143-y

[cre263-bib-0009] Hopkins, W. G. (2000). Measures of reliability in sports medicine and science. Sports Medicine, 30(1), 1–15. 10907753 10.2165/00007256-200030010-00001

[cre263-bib-0010] Lexell, J. E. , & Downham, D. Y. (2005). How to assess the reliability of measurements in rehabilitation. American Journal of Physical Medicine & Rehabilitation, 84(9), 719–723. 16141752 10.1097/01.phm.0000176452.17771.20

[cre263-bib-0011] Logemann, J. A. (1998). In Logemann J. A. (Ed.), Disorders of deglutition. In: Evaluation and treatment of swallowing disorders (pp. 44–52). Texas: Pro‐Ed Inc ISBN13:978‐0890797280.

[cre263-bib-0012] Lu, Y. M. , Lin, J. H. , Hsiao, S. F. , Liu, M. F. , Chen, S. M. , & Lue, Y. J. (2011). The relative and absolute reliability of leg muscle strength testing by a handheld dynamometer. Journal of Strength and Conditioning Research, 25(4), 1065–1071. 20838248 10.1519/JSC.0b013e3181d650a6

[cre263-bib-0013] Millwood, J. , & Fiske, J. (2001). Lip‐biting in patients with profound neuro‐disability. Dental Update, 28(2), 105–108. 11819955 10.12968/denu.2001.28.2.105

[cre263-bib-0014] Perkins, R. E. , Blanton, P. L. , & Biggs, N. L. (1977). Electromyographic analysis of the “buccinator mechanism” in human beings. Journal of Dental Research, 56(7), 783–794. 269159 10.1177/00220345770560071301

[cre263-bib-0015] Sjögreen, L. , Lohmander, A. , & Kiliaridis, S. (2011). Exploring quantitative methods for evaluation of lip function. Journal of Oral Rehabilitation, 38(6), 410–422. 20969612 10.1111/j.1365-2842.2010.02168.x

[cre263-bib-0016] Thüer, U. , & Ingervall, B. (1990). Effect of muscle exercise with an oral screen on lip function. European Journal of Orthodontics, 12(2), 198–208. 2351205 10.1093/ejo/12.2.198

[cre263-bib-0017] Yoneyama, T. , Yoshida, M. , Ohrui, T. , Mukaiyama, H. , Okamoto, H. , Hoshiba, K. , … Sasaki, H. (2002). Oral care reduces pneumonia in older patients in nursing homes. Journal of the American Geriatrics Society, 50(3), 430–433. 11943036 10.1046/j.1532-5415.2002.50106.x

